# The Use of Sensors in Blood-Brain Barrier-on-a-Chip Devices: Current Practice and Future Directions

**DOI:** 10.3390/bios13030357

**Published:** 2023-03-08

**Authors:** András Kincses, Judit P. Vigh, Dániel Petrovszki, Sándor Valkai, Anna E. Kocsis, Fruzsina R. Walter, Hung-Yin Lin, Jeng-Shiung Jan, Mária A. Deli, András Dér

**Affiliations:** 1 Institute of Biophysics, Biological Research Centre, H-6726 Szeged, Hungary; kincses.andras@brc.hu (A.K.); vigh.judit@brc.hu (J.P.V.); petrovszki.daniel@brc.hu (D.P.); valkai.sandor@brc.hu (S.V.); kocsis.anna@brc.hu (A.E.K.); walter.fruzsina@brc.hu (F.R.W.); 2 Doctoral School of Biology, University of Szeged, H-6720 Szeged, Hungary; 3 Doctoral School of Multidisciplinary Medical Sciences, University of Szeged, H-6720 Szeged, Hungary; 4 Department of Chemical and Materials Engineering, National University of Kaohsiung, Kaohsiung 81148, Taiwan; linhy@ntu.edu.tw; 5 Department of Chemical Engineering, National Cheng Kung University, Tainan 70101, Taiwan; jsjan@ncku.edu.tw

**Keywords:** biosensor, blood-brain barrier, cell surface charge, chemosensor, electrical impedance spectroscopy, optical sensor, organ-on-a-chip, streaming potential, transendothelial electrical resistance

## Abstract

The application of lab-on-a-chip technologies in in vitro cell culturing swiftly resulted in improved models of human organs compared to static culture insert-based ones. These chip devices provide controlled cell culture environments to mimic physiological functions and properties. Models of the blood-brain barrier (BBB) especially profited from this advanced technological approach. The BBB represents the tightest endothelial barrier within the vasculature with high electric resistance and low passive permeability, providing a controlled interface between the circulation and the brain. The multi-cell type dynamic BBB-on-chip models are in demand in several fields as alternatives to expensive animal studies or static culture inserts methods. Their combination with integrated biosensors provides real-time and noninvasive monitoring of the integrity of the BBB and of the presence and concentration of agents contributing to the physiological and metabolic functions and pathologies. In this review, we describe built-in sensors to characterize BBB models via quasi-direct current and electrical impedance measurements, as well as the different types of biosensors for the detection of metabolites, drugs, or toxic agents. We also give an outlook on the future of the field, with potential combinations of existing methods and possible improvements of current techniques.

## 1. Introduction

**Figure 1:**
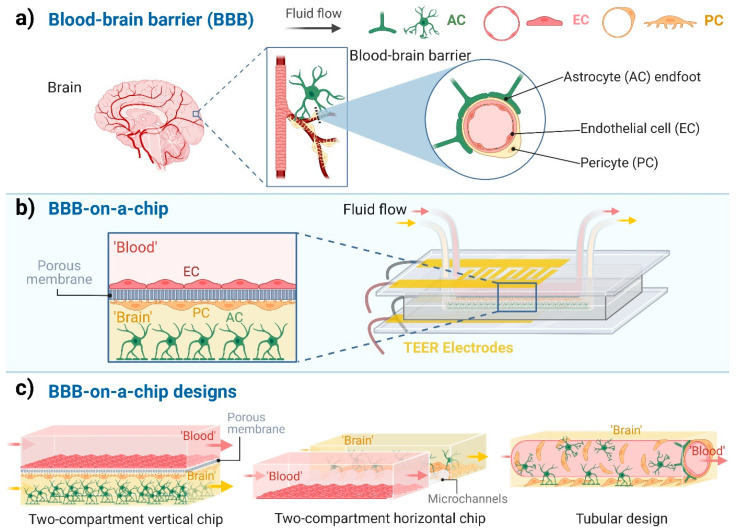
Blood-brain barrier-on-a-chip models. (**a**) The cellular composition of the blood-brain barrier (BBB). Endothelial cells (EC), which are the functional basis of the BBB, are surrounded by pericytes (PC) and the astrocytes’ endfeet (AC). (**b**) Schematic representation of a BBB-on-a-chip design with two compartments separated by a porous membrane and the co-culture of three cell types. ‘Blood’ represents the compartment with fluid flow in contact with the luminal plasma membrane of ECs. ‘Brain’ indicates the abluminal compartment in which the PCs and ACs are cultured. (**c**) Different designs of BBB-on-a-chip models. Created with BioRender.com.

The blood-brain barrier (BBB) is one the most investigated biological barriers due to its importance in different biomedical fields: these include physiological, pathological and pharmacological studies, and cover the area of central nervous system (CNS) drug delivery in health and diseases. Cultured brain endothelial cells, which have been available since the 1980s, are one of the most versatile in vitro models to examine the permeability and other properties of the BBB [1,2]. A great advancement of the BBB culture models was the introduction of co-cultures in which brain endothelial cells, which form the anatomical basis of the BBB, are kept in the presence of other brain perivascular cell types, mainly astrocytes and/or pericytes (Figure 1a), as pioneered by Nakagawa et al. in 2009 [3]. The stem cell-based BBB models introduced in the last 10 years represent a novel, improved tool for drug discovery and personalized medicine [4]. For recent reviews on BBB culture models see Helms et al. 2016 and Santa-Maria et al. 2022 [5,6]. Despite their versatility, one of the main disadvantages of the culture insert-based BBB culture models is that they are static, and do not provide shear stress, the most important mechanical force induced by fluid flow in blood vessels, necessary for vascular endothelial cell maturation.

Although dynamic BBB models using hollow fiber cartridges were introduced in the 1990s [7], in the last ten years, microfluidic chip technology has led to a breakthrough in BBB modeling (Figure 1b). These chip devices provide controlled cell culture environment to mimic physiological functions and properties. The BBB-on-a-chip (BBBoC) models can be categorized based on several parameters [8]. Most of the BBBoCs contain (at least) two compartments, one representing the blood and the other the brain tissue separated by a confluent brain endothelial layer (Figure 1b,c). These compartments can be vertically positioned, as in the culture inserts, or horizontally positioned (Figure 1c; Table 1). In some models a porous membrane separates these compartments (Figure 1b) and provide mechanical support for the brain endothelial cell layer [9], while in other models the compartments are made by hydrogels or separated by polymer scaffold [8]. A third type of BBBoCs is represented by artificially made hollow tubes in hydrogels, also called 3D tubular design (Figure 1c). Multi-compartment hydrogel-based BBBoCs were also developed to study self-organized brain microvascular networks [8]. The most common shared properties of these BBBoCs are the fluid flow in the blood compartment (Figure 1c) and the possibility to visualize the cell types in the chip devices. To characterize the paracellular tightness of the BBB models regulated by intercellular tight junctions, all these BBBoCs allow the measurement of the transfer of hydrophilic marker molecules across the brain endothelial layer [1].

**Figure 2:**
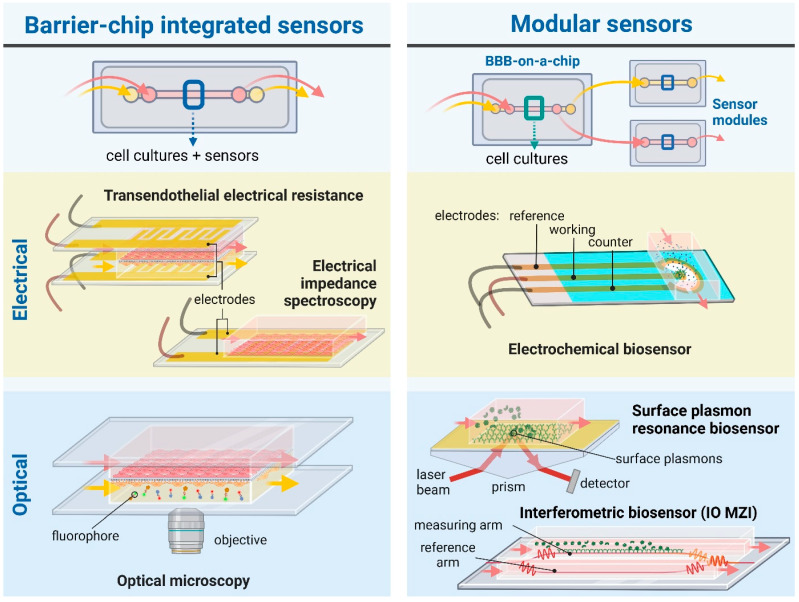
Schematic illustration of BBB-on-a-chip devices with widely used or promising integrated and/or modular (bio)sensors. For electric signal measurements, chip-integrated sensors are used to measure transendothelial electrical resistance (TEER) and electrical impedance spectroscopy, while electrochemical biosensors can be designed as modular sensing techniques. Regarding optical sensing and monitoring, microscopic observation provides a direct and practical chip-integrated approach. Evanescent-field sensing methods, such as surface plasmon resonance or integrated optical (IO) interferometry—e.g., Mach-Zehnder interferometer (MZI)—can be used as modules attached to chips to detect bioparticles, e.g., proteins, pathogens, of interest. The figure was created with Biorender.com.

The BBB represents the tightest endothelial barrier within the vasculature which is reflected by the high transendothelial electric resistance (TEER) values [10]. The combination of BBBoCs with integrated electrical biosensors was already introduced in the first prototypes to measure TEER and thus provide real-time and noninvasive monitoring of the integrity of the BBB (Figure 2) [11]. While BBBoCs with integrated and/or modular biosensors hold a great potential to monitor the presence and concentration of agents contributing to the physiological and metabolic functions and pathologies of the BBB, most of the integrated BBBoC sensors measure electrical or electrochemical signals (Figure 2).

In this review, we describe current built-in sensors to characterize BBBoC models via quasi-direct current (DC), electrical impedance spectroscopy (EIS) and electrochemical measurements. Since all the available BBBoCs containing biosensors belong to the designs shown in Figure 1c, we focused only on these models, and other models like the self-organized brain microvascular networks were out of the scope of the present review. We also give an outlook on the future of the field, with potential combinations of BBBoCs with different types of biosensors for the detection of metabolites, drugs, or toxic agents that can further improve the power of this novel technology.

## 2. The Evaluation of the BBB Integrity Based on Electrical Parameters

The passive electrical properties of the in vitro BBB models provide structural information about the barrier, including the integrity of the endothelial monolayer and cell morphology. The lipid bilayers and thus the cells act as electrical insulators, therefore the intercellular tight junctions primarily dominate the passive electrical properties [10]. TEER and EIS are the standard methods used to characterize BBB models in static culture inserts; for a review see Vigh et al. 2021 [10]. These non-invasive, real-time and fast measurements were naturally applied in the first chip devices as well. Since microfluidics and photolithographic technologies became available in more and more laboratories, the use of integrated electrodes in BBBoC devices became a standard and well-established technique in the past decade (Figure 2). The shape and material of the electrical sensors mainly depend on the application and the channel geometries of the BBBoCs (Table 1). The general considerations are the good and stable electrical properties and the biocompatibility of the electrodes. Most devices use platinum, gold or silver/silver chloride as materials of the electrodes. The first two have superior conductivity, but the electrical potential of the interface is less stable, thus results depend on the environment. It is still possible to overcome the instability with the proper experimental setting. On the contrary, silver and silver chloride have great stability, especially when there is no current flow through the electrodes. Some BBBoCs use wire electrodes, others are equipped with deposited film metal electrodes (Table 1). An advantage of the wire electrodes is that these do not need any special laboratory equipment and cleanroom facilities. They are used in cases where local sampling of the electrical potential or current is sufficient. However, a precise characterization of the overall conductivity of a planar brain endothelial layer usually requires more extensive integrated electrodes, which can be produced via sputter coating. The deposited electrodes are created on the solid surfaces of the channels with well-defined size, shape and transparency, either with masking or with lift-off techniques. These methods require more complex laboratory instrumentation.

### 2.1. Trans-Endothelial Electrical Resistance Measurements

The DC and quasi-DC methods measure the ohmic resistance of the brain endothelial cell layer. The most direct approach in this case is to place a pair of electrodes on each side of the cell culture membrane (Figure 1b). Then, the ohmic resistance is calculated based on the applied current and the measured voltage via Ohm’s law [8,10]. However, the electrode-electrolyte interface limits the applicability of the two-electrode measurements in the case of DC (and at low frequency). Four-point measurement systems are applied to avoid electrode polarization, where one pair of the electrodes inject current through the cell layer and the other pair measure the generated voltage (Figure 1b and Figure 2). In this case, the net resistance is also calculated via Ohm’s law. Another possibility for the evaluation of the barrier integrity is the EIS (Figure 2). A sinusoidal electric signal is applied on the sample, generally in the Hz to a few hundred kHz region, hence the mobile ions are displaced according to the direction of the electric field. The charge displacement happens alongside the charged cell membrane surfaces. The charge displacements are followed by energy dissipation, so the sample can be characterized with a complex dielectric permittivity. The real part describes the polarization (capacitance) and the imaginary part describes the dissipation (resistance). Thus, EIS gives more detail about the barrier integrity as compared to TEER. However, TEER needs cheaper and simpler instrumentation. Despite these slight differences, both methods provide rapid, real-time information about the barrier integrity, which is extremely important in the case of the BBB models [10]. A wide range of BBBoCs utilize TEER and EIS sensors for monitoring the overall tightness or disruption of the barrier integrity (Table 1).

**Table 1:** Measurement of barrier integrity and cell surface charge of blood-brain barrier models on chip devices with electrical sensors.

Chip Type	Electrode/Measuring Instrument	BBB Model	Reference
**Trans-endothelial electrical resistance**			
two-compartment vertical chip	4 Ag/AgCl film electrodes, EVOM-2 (WPI)	bEnd.3 mouse brain endothelial & C8D1A astrocyte cell lines	[11,12]
two-compartment vertical chip	4 Au film electrodes, EVOM-2 (WPI)	hCMEC/D3 human brain endothelial cell line co-culture: primary rat brain endothelial cells, brain pericytes, astrocytes	[9]
two-compartment vertical chip	4 Au film electrodes, EVOM-2 (WPI)	co-culture: human SC derived endothelial cells, brain pericytes	[13]
two-compartment vertical chip	4 Ag/AgCl wire electrode, ERS (Millicell)	co-culture: iPSC derived endothelial cells, rat astrocytes	[14]
two-compartment vertical chip	4 Ag/AgCl wire electrodes, EVOM-2 (WPI)	bEnd.3 mouse brain endothelial cell line	[15]
two-compartment vertical chip	4 × 4 Au film MEA electrodes, EVOM-2 (WPI)	co-culture: primary mouse brain endothelial cell, astrocytes	[16]
two-compartment vertical chip	Au film electrodes	co-culture: iPSC derived endothelial cells, pericytes, astrocytes	[4]
two-compartment vertical chip	4 Ag/AgCl wire electrodes, EVOM-2 (WPI)	co-culture: human HBMEC brain endothelial cell line, brain pericytes, astrocytes	[17]
two-compartment horizontal chip	2 Ag/AgCl wire electrodes, EVOM-2 (WPI)	co-culture: primary rat brain endothelial cells, astrocytes	[18]
three-compartment horizontal chip	2 Pt film electrodes, EVOM-2 (WPI)	co-culture: HUVECs, human astrocytes	[19]
parallel tubular channel horizontal chip	wire electrodes, ERS (Millicell)	bEnd.3 mouse brain endothelial cell line	[20]
**Electrical impedance spectroscopy**			
two-compartment vertical chip	2 Pt wire electrodes, HP4194A impedance analyzer (Hewlett-Packard)	hCMEC/D3 human brain endothelial cell line	[21]
two-compartment vertical chip	4 Pt film electrodes, custom impedance analyzer with AD5933 chip (Analog Devices)	co-culture: primary human brain endothelial cells, pericytes, astrocytes	[22,23]
two-compartment vertical chip	4 Pt wire electrodes, SP-300 potentiostat (Bio-Logic Science Instruments), HP4194A impedance analyzer (Hewlett Packard)	hCMEC/D3 human brain endothelial cell line	[24]
two-compartment vertical chip	2 Pt wire electrodes, PGSTAT302N potentiostat with FRA32M frequency response analysis module (Metrohm Autolab BV)	bEnd.3 mouse brain endothelial cell line	[25]
two-compartment vertical chip	4 Au film electrodes, PGstat128N (Metrohm Autolab BV)	co-culture: human iPSC derived endothelial cell, human brain pericytes, astrocytes	[26]
two-compartment vertical chip	2 Ag/AgCl wire electrodes, E4980AL/032 LCR meter (Keysight Technologies)	co-culture: human iPSC derived endothelial cell, human astrocytes	[27]
two-compartment vertical chip	4 Pt wire electrodes, HF2IS impedance spectroscope, HF2LI lock-in amplifier (Zurich Instruments)	hCMEC/D3 human brain endothelial cell line	[28]
two-compartment horizontal multiplexed chip (Organoplate)	stainless-steel multiplexed pair-electrodes, MI-OT-1 OrganoTEER device (MIMETAS)	co-culture: human primary brain endothelial cells, astrocytes, iPSC derived neurons	[29]
parallel tubular channel horizontal chip	2 wire electrodes, Stingray DS1M12 USB oscilloscope and signal generator (USB Instruments)	co-culture: hCMEC/D3 human brain endothelial cell line, human astrocytes	[30]
**Streaming potential**			
two-compartment vertical chip	2 Ag/AgCl wire electrodes, SR560 voltage pre-amplifier (Stanford Research Systems), Wave Ace digital oscilloscope (Teledyne LeCroy)	hCMEC/D3 human brain endothelial cell line	[31]

Abbreviation: ERS, electrical resistance system; EVOM, Epithelial Voltohmmeter; HUVEC, human umbilical vein endothelial cells; iPSC, induced pluripotent stem cell; MEA, multi-electrode array; SC, stem cell; WPI, World Precision Instruments.

The first BBBoC, the μBBB was reported by Booth and Kim in 2012 and in a follow-up paper in 2014 [11,12]. In this device a pair of perpendicularly-crossing channels was separated by a porous polycarbonate membrane. The brain endothelial cells (bEnd.3 mouse cell line) and the astrocytes (C8D1A mouse cell line) were seeded in the top and bottom channels, respectively. The channels were covered with glass slides containing the TEER sensors. The top channels were connected to a peristaltic pump to mimic the shear stress of the blood flow. The three metal layers of Cr-Au-Ag of the four-electrode measurement system was fabricated by sputter deposition, then Ag layers were chlorinated chemically with FeCl_3_. The four silver/silver chloride electrodes were connected to a commercially available measurement device, EVOM2 endothelial voltohmmeter (Table 1). The EVOM2 applies a quasi-DC 12.5 Hz square signal during measurement. The TEER values were calculated by extracting the background resistance from the total at each case, and normalized for the area of the culture surface with the following equation:

$$TEER=\left(R_{t}-R_{bg}\right)\times A$$

where *R_t_* is the total resistance, *R_bg_* is the background and *A* is the culture surface. Despite the low shear stress, the *TEER* values in the dynamic μBBB was more than ten times higher than in the static culture inserts [11].

Walter et al., reported a versatile organ-on-a-chip device that was used for the characterization of two different BBB culture models [9]. The two parallel channels were separated by a porous polyethylene membrane. The integrated gold electrodes monitored the whole culture area (2 mm × 36 mm) and provided 4-point TEER measurements. The thickness of the sputter-coated gold film was set to 25 nm, so the layer was conductive, while it was still transparent. The great advantage of this type of film electrodes is that the measured TEER values characterize the whole culture surface and the cell monolayer could still be monitored with optical microscopy along the entire channel [9]. Two BBB models were tested in the device under static and dynamic flow conditions: the human hCMEC/D3 brain endothelial cell line and a triple co-culture model of primary rat brain endothelial cells, brain pericytes and astrocytes. The endothelial cells and the pericytes were cultured on the top and bottom sides of the membrane, and astrocytes at the bottom of the lower culture channel, respectively (Figure 1b). The *TEER* values, calculated as described above, increased significantly under flow conditions compared to the static circumstances [9].

### 2.2. Electrical Impedance Spectroscopy

The other group of BBBoC devices use EIS for the characterization of the BBB integrity (Table 1). Such device was reported by Griep et al. with two perpendicularly crossing PDMS channels [21]. Polycarbonate culture membrane was inserted between the top and bottom channels at the overlapping central section as a support for the hCMEC/D3 brain endothelial cell line. A pair of platinum wires served as electrodes for the EIS measurements. The amplitude of the AC was 10 mV and a wide frequency range were swept from 1 Hz to 3 MHz with the SP-300 potentiostat (BioLogic, Seyssinet-Pariset, Auvergne, France). The TEER values were measured at 10 kHz frequency. Another EIS based BBBoC device with similar structure was published by van der Helm et al. in 2016 [24]. Helm’s device also had two X-shaped PDMS channel, but the main difference was that there were four platinum wires instead of two. The impedance spectra were recorded in two-electrode configuration with a homemade device (validated by SP-300 potentiostat, BioLogic and Impedance Gain/Phase analyser, Hewlett Packard) from 200 Hz to 1 MHz to each electrode combination. Similar to Griep’s results, the resistive plateau was found at 10 kHz and the *TEER* was calculated from the results of each pairing via Gaussian elimination:

$$TEER=A_{cult}\times R_{m}=A_{cult}\times \frac{1}{4}\left(R_{1-2}+R_{1-4}+R_{2-3}+R_{3-4}-2R_{1-3}-2R_{2-3}\right)$$

where *R_m_* is the net resistance of the monolayer and *R_i_*_-j_ were the resistances between the electrodes.

Most of the BBBoC devices provide 2D cell culture surfaces, which is convenient for several reasons, such as cell visibility by microscopy, TEER measurement, and permeability assays. However, it is obvious that a 3D tubular surface represents a more physiological-like structure. Dynamic in vitro BBB models using hollow fiber cartridges were introduced well before 3D vascular chip models [7], but their use was rather limited and few of them were equipped to measure TEER [32]. Partyka et al. overcame this issue with a hydrogel-based 3D tubular BBBoC with the possibility to measure TEER [30]. The base of the chip, including the parallel channels and inlets/outlets, was prepared with soft lithography using PDMS. The central section of the channels was embedded in hydrogel, which contained the astroglia cells, while the hCMEC/D3 brain endothelial cells were seeded into the hydrogel tubes. This transparent region made possible the optical monitoring of the cell growth via bright-field imaging, confocal microscopy and TEM. The electrodes were placed at the inlet ports and EIS was performed using a Stingray DS1M12 USB oscilloscope adapter (Table 1). The frequency range of the applied voltage was swept from 15 Hz to 15.6 kHz. The impedance was calculated by the ratio of the amplitudes of the applied voltage and measured current. The net TEER values represented the difference between the impedances at 15 Hz and 15.6 kHz. The impedance of the former was dominated by the capacitance of the electrodes, while the main component of the latter was the ohmic resistance of the culture medium.

## 3. Surface Charge Determination—Streaming Potential Measurement

The importance of brain endothelial surface charge in the context of BBB physiological functions, pathologies and CNS drug delivery is getting more and more attention [33]. It is well-known that the luminal surface charge of the endothelium of the BBB is low compared to other endothelial cell types [34]. Typically, zeta potential is measured on cell suspensions [34], rather than on monolayers. Kincses at al presented a BBBoC device for streaming potential-based characterization of the surface potential of brain endothelial cell monolayers [31]. The chip was based on a previously reported device by Walter et al. [9], which was upgraded with a pair of Ag/AgCl electrodes at the inlet and outlet of the top channel containing the brain endothelial cell layer (Table 1). For the streaming potential measurements, the flow was periodically stopped and restarted. After each restart, a transient electrical signal was recorded using a voltage amplifier (SR 560, Stanford Instruments) and an oscilloscope (Wave Ace, Teledyne LeCroy, Chestnut Ridge, NY, USA). The source of the transient signal was the mobile ions of the Guy-Chapman double layer above the charged cell surface, which was grabbed by the flow and accumulated at the large vicinity of the outlet electrode. The amplitude of the transient signal was proportional to the zeta potential of the monolayer. The results were validated via COMSOL Multiphysics simulations and control experiments on cell suspension with laser-Doppler velocimetry [31].

## 4. Other Current and Potential Sensor Types for BBB-on-a-Chip Platforms

**Figure 3:**
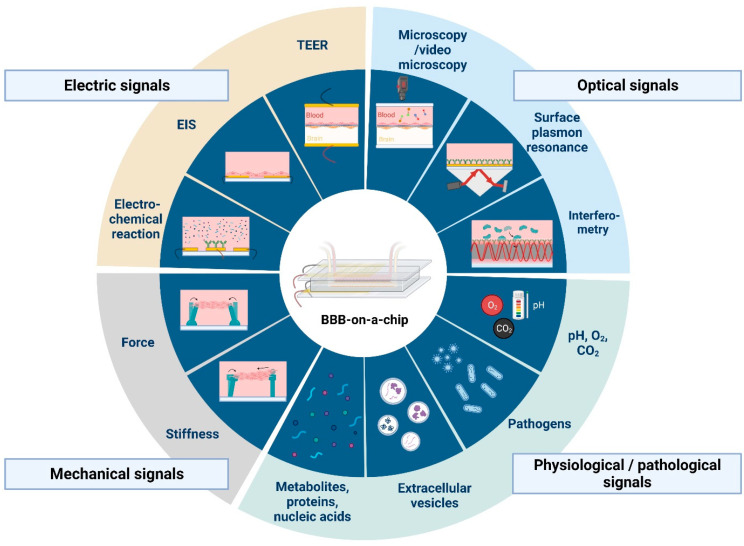
Current and potential sensing technologies that can be integrated with blood-brain barrier-on-a-chip devices. Abbreviations: TEER, transendothelial electrical resistance; EIS, electrical impedance spectroscopy. Created with BioRender.com.

There are several applications of sensors and biosensors for the evaluation of metabolic activity in organ-on-a-chip devices. These have different working principles; we can find amperometric, potentiometric, electrochemical or optical sensors (Figure 2 and Figure 3). Such sensors can monitor O_2_ concentrations, pH of the culture medium or the amount of certain metabolites, like glucose or lactate, as well as the presence of biomarker proteins or pathogens [35,36]. However, the application of sensors in BBBoC devices is still very scarce, and space restrictions can be one of the reasons. With the latter in mind both integrated and modular sensors can be designed for BBBoCs (Figure 2).

## 5. Electrochemical or Optochemical Oxygen Sensors

One of the few examples of other types of sensors in BBBoC are two non-invasive real-time oxygen biosensors (Figure 3) with tunable oxygen scavenging material, thiol-ene-epoxy [37]. The BBBoC model under low oxygen levels represented a pathologically relevant example of hypoxic conditions during ischemic stroke. Two complementary oxygen sensors were applied to monitor oxygen levels in the microchannels. One was an opto-chemical oxygen sensor based on luminescent lifetime measurement, which used conjugate microplates of platinum and palladium indicator dyes [37]. The other sensor used an electrochemical method, and detected dissolved oxygen levels via oxygenation at the surface of a Clark-electrode. Both optical and electrochemical sensing methods showed the same oxygen depletion trends, with initial fast scavenging rates followed by a slower second linear. The thiol-ene-epoxy-based chip could be used for longer follow-up of the culture conditions, since it maintained the oxygen-scavenging properties throughout an 18-day period [37].

## 6. Chemosensors with Molecularly Imprinted Polymers

Molecularly imprinted polymers (MIPs) are polymerized in the presence of a guest template, and thus (when template is removed) they present cavities or depressions that are complementary in size and functionality to the template and can rebind it non-covalently [38]. The affinities so far achieved do not compare with natural antibodies, but MIPs are nonetheless finding important uses in bioseparations [39,40,41,42], biosensing [43,44,45], biocatalysis [46], and drug delivery [40,47].

MIPs in diagnostics have been recently reviewed that allow high-affinity analyte detection in various biological fluids, namely serum, saliva, cerebrospinal fluid, sweat, urine, nasopharyngeal fluid, and tears [48]. Our previous work also demonstrated the measurements of many biomarkers with MIPs in urine [45], saliva [49], and serum [50]. The imprinting templates were from the whole proteins to the peptides of the target molecules, such as albumin, lysozyme [44], ribonuclease A and myoglobin [43], matrix metalloproteinase 1 [51], C-reactive protein [50], and α-synuclein (SNCA) [52]. Moreover, antibodies against toxic disease proteins are developed as possible therapeutics for including Parkinson’s disease [53]. Our recent work described the preparation and characterization of peptide-imprinted composite nanoparticles that not only allowed the detection of SNCA but also the extraction of SNCA from CRISPR/dCas9a-activated HEK293T cells [42]. MIP-based chemosensors [54] could be valuable in detecting the transfer, influx or efflux of various things—e.g., bioactive molecules, such as dopamine; CNS pathological factors, such as β-amyloid oligomers; serum proteins, such as albumin; and pathogens, such as viruses—across brain endothelial cell layers using BBBoCs. This type of sensor can be potentially also be used in pathological conditions to monitor the shedding of glycocalyx elements, such as sialic acid [54] from the luminal surface of brain endothelial cells, or to detect in the “blood” compartment markers of BBB leakage originating from the “brain” compartment, such as astrocyte markers glial fibrillary acidic protein and S100B, or neuron-specific enolase.

## 7. Optical Biosensors as Promising Candidates for Incorporated Sensing and Monitoring

Optical monitoring of physiological parameters, biomolecular interactions and changes in barrier integrity can also provide valuable information about the microenvironment of the BBB models (Figure 2 and Figure 3). For this purpose, labeling techniques, including fluorophores or biochemical interactions with fluorescent products, are widely used to observe in vitro cell culture systems by microscopic techniques or optical biosensors [55,56,57].

A good example of the microscopic technique is a BBBoC with integrated digital immunosensors to determine cytokine release, as described in a recent paper [58]. Named DigiTACK, this device allowed the sequential multiplexed cytokine profiling of primary mouse brain endothelial cells grown as monolayers on a nanoporous silicon nitride membrane. The luminal and basal levels of interleukin-6, CCL2/MCP-1 and CXCL1/KC cytokines were detected at three time-points using a digital immunosensor signal readout process by fluorescent microscopy [58].

The application of chip-integrated optical biosensors (Figure 2) is a promising alternative able to monitor biological events or detect a wide range of analytes. Among these devices, integrated optical ones are promising in terms of miniaturization, portability, sensitivity, specificity, rapid operation and, last but not least, their ease of integration with microfluidics. Due to these favorable properties, numerous label-free, integrated optical biosensors are presented and widely investigated in the field of point-of-care diagnostics [59]. An extensively used sensing technique among such sensors is evanescent-field sensing, which gives the basis of various sensing devices. This working principle enables the detection of the target analytes in the microenvironment of a waveguiding structure in a few hundred nanometers in the vicinity of the sensor waveguide structure, based on the change in the local refractive index, produced by biomolecular interactions. By functionalizing the surface of the guide, specific detection could be performed as well.

In an interferometric construction (Figure 2), this approach could be very sensitive. Therefore, several integrated optical interferometers have been realized for the detection of pathogens, such as viral particles or bacteria, thus the applicability of this technique has been well proved for point-of-care diagnostic purposes. In a recent study, Petrovszki et al., applied such an optical biosensing approach to examine the barrier penetration capability of the surface spike protein subunit 1 (S1) of SARS-CoV-2 across culture models of the BBB and the intestinal barrier [60]. After performing the permeability assays, the amount of the target proteins which could cross the corresponding barrier model was evaluated by using an integrated optical Mach-Zehnder interferometer biosensor. As a result of the optical biosensing approach, a difference was found in the S1 spike protein passage for the different cell culture models: S1 could cross the human brain endothelial barrier better than the intestinal barrier. The results obtained from this biosensing approach were in accordance with the ones measured in parallel with specific ELISA [60].

Another label-free, interferometric integrated optical biosensor construction is the bimodal waveguide interferometer, which technology has emerged in recent years. These devices have a small footprint and their performance has been demonstrated in the case of several target detection from fluid samples, e.g., microRNA marker [61], SARS-CoV-2 viral proteins [62], and bacteria [63,64]. The above-mentioned studies clearly show the impact of the application of such integrated optical interferometric biosensors in chip devices as incorporated sensors. Their good performance, label-free operation and easy integration with microfluidics support such a perspective.

## 8. Mechanical Signal Detection

Mechanical properties of cells comprising the BBB play a fundamental role in determining the adaptability and vulnerability of the barrier subjected to external stimuli. The most commonly used methods of mapping the stress and strain fields associated to cell layers adapt one form or another of traction force microscopy, where the cells are grown on the surface of an elastic material (e.g., hydrogel or PDMS) doped with fluorescent microbeads or equipped with a micropillar-brushed surface [65]. If, however, only the overall principal components of the stress tensor are necessary to obtain, conductivity measurements across the elastic substrate doped with carbon nanotubes can also be used [66]. Such sensors (Figure 3) have not been applied in BBBoCs yet. The dilation and contraction of cerebral microvessels is linked to brain activity-dependent blood flow regulation known as neurovascular coupling [67]. Both brain pericytes at the level of brain capillaries and smooth muscle cells at the level of microvessels are contractile. The measurement of dilation or contraction of brain pericytes, perivascular smooth muscle cells or brain microvessel mimics with tubular design (Figure 1) can be important in studying pericyte interactions with other BBB cells and neurovascular coupling in physiological or pathological conditions.

## 9. Future Perspective

The ultimate goal of BBBoC developers is to provide a complex platform including both the physiologically relevant barrier model system and the tools to monitor its functional properties (Figure 2 and Figure 3). Despite recent advances in microfabrication techniques [68,69,70,71,72,73,74,75,76], to date this still remains a very challenging task, mainly due to space limitations and the disturbing interference of the operation of different sensing elements. In order to circumvent these problems, the spatial separation of various monitoring tasks seems to be inevitable.

Real-time characterization of the most relevant physical parameters requires direct access to the BBB model via electrical sensors measuring TEER and zeta-potential for the transmembrane conductivity and surface electric charge, respectively, or by various imaging techniques, such as phase-contrast or fluorescent microscopy [77]. In contrast, chemical and biochemical signals can be monitored at separate sites, coupled with the main BBB module via microfluidic channels [35,78]. An interconnected microfluidic network of (bio)sensors is envisioned to be created this way (Figure 2), where the spatio-temporal organization of the fluid flow can be controlled by programmable valves [73,79]. Such complex sensing platforms are expected to provide high versatility and flexibility for the scientific investigation of BBB culture models, as well as for targeted applications in point-of-care diagnostics.

While at present, integrated sensors in BBBoCs measuring anything other than electrical signals are restricted to detecting oxygen [37] and cytokines [58], there is an urgent need to integrate further biosensors into future BBB-model platforms that are capable to detect other substances as well. Molecules of various size and electric charge used for permeability assays, such as labeled dextrans, rhodamine dyes, antibodies or albumin, should be routinely measured by integrated biosensors in the future. Penetration of proteins and cells associated to pathologies, such as viral proteins or cancer cells, respectively [60,70], or transport of potential pharmacons, alone or bound to carrier molecules or nanoparticles, should also be quantitatively characterized [80]. Brain endothelial cells are known to secrete extracellular vesicles to both the basal and apical sides in normal physiological homeostasis, while vesicle secretion rapidly increases under inflammatory conditions [81]. An important new branch of studies investigating the concentration and composition of extracellular vesicles is emerging [82], where relevant biosensors capable of real-time monitoring are expected to give a high added value.

Recent developments in microfluidic technology also accomplished matching of the most powerful mass spectroscopic method, inductively coupled plasma mass spectrometry (ICP-MS), with chip devices. This paves the way to a determination of trace elements or stable isotopes in biological samples, allowing the ultrasensitive chemical analysis of the fluidic environment of BBB model systems as well [83].

The method of detection used in biosensors depends, amongst other factors, on the size of the analyte. Integrated optical sensors based on evanescent-wave and label-free detection are of extremely high sensitivity for a wide size range, from large cells down to proteins or other macromolecules [84,85,86,87,88,89,90]. Although their application usually requires a separate laser source, given the recent technical advances in miniature diode-laser development, it does not represent a practical problem when using a modular biosensor arrangement. The specificity of the detection in this case is provided by targeted functionalization of the sensor surface (usually that of a waveguide by antibodies or aptamers), and adsorption of analyte molecules in the evanescent field results in modification of the phase-modulation of the guided light that can be translated to intensity or spectral changes, depending on the type of the detector [86,91,92]. An interesting approach is using functionalized gold (or silver) nanobeads, whose SPR-determined absorption spectra suffer a wavelength-shift upon adsorption of the analyte molecules [93]. Microfluidics allows a rapid exchange of the beads after the measurement is completed [94], thereby regenerating the biosensor’s sensitivity (Figure 2), which circumvents inactivation of the sensor, which represents a serious problem in the case of other surface-sensitive detection techniques.

For the detection of small molecules, electrical and electrochemical biosensors seem to be the optimal choice. They are cheap, easily integrable into chip devices, and allow rapid sensing. Depending on the particular problem, potentiometric, amperometric, voltammetric or field-effect transistor-based biosensors can be chosen [95,96,97,98,99], while recent studies have also utilized the advantages of electro-chemiluminescent detection in various bioanalytical applications [100]. In addition, both electric and optical biosensors can make use of biocompatible smart materials, such as graphene oxide derivatives [101], which can be functionalized by molecular grafting techniques [102,103]. The application of biosensors utilizing the synergistic interaction of electric and optical phenomena can also be advantageous in BBB research, especially when the concentration of penetrating cells or secreted extracellular vesicles are supposed to be quantified [60]. For the rapid separation of extracellular vesicles according to size, simple filter-based chip modules appear to be the optimal choice [104], while their proteomic and genomic analysis can be carried out with coupled sensing or amplifying units [105,106]. An interesting new concept suggests the use of in vitro companion biomarker diagnostic devices through all the phases of drug development from preclinical tests to clinical studies in Alzheimer’s disease precision medicine [107]. BBBoCs with patient-derived cells and biosensors could be especially useful in such drug research and development scenario to promote a more patient-centric approach.

All in all, modular networks of microfluidic BBBoCs and biosensors are expected to be developed in the near future for both basic- and applied-science utilization. Online control and measurement techniques will provide multiplex time series of data carrying independent information about the barrier properties. For the analysis of such complex data streams, artificial intelligence methods are expected to be especially advantageous [102,104].

## Data Availability

Not applicable.
